# Post-transcriptional regulation via alternative polyadenylation and piRNA shapes macrophage responses in psoriasis

**DOI:** 10.3389/fimmu.2026.1760348

**Published:** 2026-07-23

**Authors:** Benfan Lin, Xiwang Du, Leihong Lu, Zongshan Wang, Yan Zhao, Yue Feng, Liqian Liu

**Affiliations:** 1 Department of Dermatology, Linyi People’s Hospital, Linyi, China; 2 Department of Dermatology, General Hospital of Fushun Mining Bureau of Liaoning Health Industry Group, Fushun, China; 3 Department of Femoral Head, Linyi People’s Hospital, Linyi, China; 4 Special Inspection Department, Shandong Cancer Hospital and Institute, Shandong First Medical University and Shandong Academy of Medical Sciences, Ji’nan, China

**Keywords:** alternative polyadenylation, macrophage heterogeneity, piRNA, post-transcriptional regulation, psoriasis, TDRKH

## Abstract

**Background:**

Post-transcriptional regulatory mechanisms underlying autoimmune diseases remain poorly understood. Here, we employed integrated multi-omics approaches to systematically dissect the role of alternative polyadenylation (APA) and piRNA-mediated regulation in psoriasis pathogenesis.

**Methods:**

We performed transcriptome-wide association studies (TWAS) combined with Mendelian randomization to identify causal susceptibility genes. Long-read sequencing was used to characterize APA landscapes in 15 healthy controls and 10 psoriasis patients. Single-cell RNA sequencing was performed on lesional tissues from 4 patients to define cell-specific expression patterns. Small RNA sequencing was conducted to profile piRNA regulation in LPS + IFN-γ–polarized THP-1 macrophages following TDRKH knockdown.

**Results:**

Integrating multi-tissue genetic validations across GTEx, eQTLGen, and FinnGen datasets, we identified TDRKH as a robust psoriasis susceptibility gene (PPH4 > 0.92). TDRKH exhibited significant APA alterations in psoriatic PBMCs, with preferential usage of the distal polyadenylation site leading to 3′UTR lengthening (PDI = −0.574). Reporter gene assays confirmed that the long 3′UTR isoform conferred approximately 3-fold greater mRNA stability (t½ = 6.9 h vs. 2.3 h). Single-cell RNA sequencing of psoriatic skin (4 patients, 17,734 cells) identified 17 cell types and 10 macrophage/myeloid subpopulations using marker gene-based annotation with TDRKH excluded from feature selection. TDRKH expression was highest in IL-23-producing macrophages (IL23A+IL1B+), which were selectively enriched in lesional tissue. TDRKH knockdown in LPS + IFN-γ–polarized THP-1 macrophages altered piRNA expression profiles, reduced pro-inflammatory surface markers (CD80, CD86, HLA-DR), and attenuated IL6 and IL1B expression, supporting a functional role of the TDRKH–piRNA axis in macrophage inflammatory activation.

**Conclusions:**

Our findings reveal a previously unrecognized post-transcriptional regulatory network involving APA and piRNA that governs macrophage-driven inflammation. The discovery of the TDRKH-piRNA axis provides novel mechanistic insights into psoriasis and establishes a potential therapeutic target.

## Background

Psoriasis is a common chronic inflammatory skin disease affecting 2-3% of the global population, imposing significant physiological and psychological burdens on patients (1). The disease is pathologically characterized by epidermal hyperproliferation, inflammatory cell infiltration, and neovascularization, clinically manifesting as well-demarcated erythematous plaques covered with silvery-white scales (2). Although the advent of biological therapies has substantially improved treatment outcomes in recent years, a considerable proportion of patients remain refractory to existing treatments or develop secondary treatment failure, indicating that our understanding of psoriasis pathogenesis remains incomplete (3).

Psoriasis demonstrates strong genetic predisposition, with concordance rates reaching 70% in monozygotic twins and first-degree relatives exhibiting a 10-fold increased risk compared to the general population (4). Over the past two decades, genome-wide association studies (GWAS) have identified more than 80 psoriasis susceptibility loci, encompassing multiple biological pathways including innate immunity, adaptive immunity, and skin barrier function (5). However, these genetic variants explain only approximately 30% of disease heritability, with most variants located in non-coding regions whose functional mechanisms remain elusive (6). More critically, the molecular mechanisms bridging genetic variants to disease phenotypes remain a “black box,” limiting the translation of genetic discoveries into clinical applications.

To address these limitations, transcriptome-wide association studies (TWAS) have emerged as a powerful approach that integrates GWAS data with expression quantitative trait loci (eQTL) information to identify disease-associated gene expression alterations, thereby complementing traditional GWAS in functional interpretation (7). Furthermore, summary-data-based Mendelian randomization (SMR) methods employ causal inference frameworks to distinguish genuine causal genes from false-positive signals arising from linkage disequilibrium (8). The combined application of these methodologies provides robust tools for dissecting the genetic architecture of complex diseases.

Beyond the effects of genetic variants on gene expression levels, post-transcriptional regulation has gained increasing recognition for its role in disease pathogenesis. Alternative splicing (AS) and alternative polyadenylation (APA) represent two major post-transcriptional regulatory mechanisms that significantly expand transcriptomic and proteomic diversity without altering gene copy numbers (9, 10). In inflammatory diseases, dysregulation of these mechanisms has been demonstrated to modulate immune cell activation, differentiation, and effector functions (11). The development of third-generation sequencing technologies has enabled direct acquisition of full-length transcript information, providing unprecedented opportunities for systematic investigation of post-transcriptional regulation (12).

PIWI-interacting RNAs (piRNAs) constitute a class of small non-coding RNAs ranging from 24–32 nucleotides, initially discovered in germline cells where they primarily maintain genomic stability (13). Recent evidence increasingly indicates that piRNAs are also expressed in somatic cells and participate in various physiological and pathological processes (14). Particularly within the immune system, piRNAs have been shown to regulate macrophage activation and inflammatory responses (15); however, their mechanistic roles in autoimmune diseases remain poorly understood.

Macrophages serve as key effector cells in psoriasis pathogenesis, initiating and maintaining the IL-23/IL-17 inflammatory axis through secretion of cytokines including IL-23 and TNF-α (16). While macrophages were historically classified into pro-inflammatory (M1) and anti-inflammatory (M2) subsets, it is now recognized that macrophage activation exists along a multidimensional spectrum of functional states rather than a binary dichotomy (17–20). Single-cell transcriptomic studies have revealed that tissue macrophages adopt diverse, context-dependent activation programs that do not conform to the classical M1/M2 framework (21). In psoriatic lesions, macrophages exhibit marked functional heterogeneity, with subsets expressing distinct inflammatory mediators and surface markers; however, the molecular mechanisms governing this heterogeneity and its contribution to disease pathogenesis remain unclear (22).

The application of single-cell RNA sequencing technology has revolutionized our understanding of cellular heterogeneity within tissue microenvironments. In psoriasis research, single-cell analyses have revealed multiple functionally distinct immune cell subpopulations within lesions and their complex interaction networks (23). This high-resolution analytical approach enables investigation of gene expression regulation at the single-cell level, facilitating identification of disease-associated cell subpopulations and molecular markers.

Based on this comprehensive background, the present study aims to systematically elucidate the genetic susceptibility mechanisms and molecular regulatory networks underlying psoriasis through integration of multi-layered genomic and transcriptomic data. We first employ TWAS and SMR methods to identify causally associated genes based on large-scale GWAS data. Subsequently, we utilize third-generation sequencing technology to comprehensively characterize post-transcriptional regulatory features of these candidate genes. We further apply single-cell RNA sequencing to resolve cell-specific expression patterns of key genes within the lesional microenvironment. Finally, we validate core regulatory mechanisms through functional experiments. Through this multi-omics integration strategy, we expect to construct a complete regulatory cascade from genetic variants to disease phenotypes, thereby providing novel theoretical foundations and potential therapeutic targets for precision medicine in psoriasis.

## Methods

### Data sources and study design

This study employed a multi-layered integrative analysis strategy, combining genetic, transcriptomic, and single-cell genomic approaches to systematically investigate the pathogenic mechanisms of psoriasis. Gene expression data were obtained from the Genotype-Tissue Expression (GTEx) project version 8, including non-sun-exposed skin tissue and whole blood tissue, as well as blood eQTL summary data from the eQTLGen consortium. Psoriasis GWAS data were sourced from the FinnGen database.

### Transcriptome-wide association study

Genome-wide TWAS analysis was performed using FUSION software (version 2019-07-19). This method integrates tissue-specific eQTL weights with GWAS summary statistics to predict the impact of gene expression on disease risk. The analysis encompassed all protein-coding genes across 22 autosomes, using the 1000 Genomes Project European population as a reference panel for linkage disequilibrium calculations. Bonferroni multiple testing correction was applied, with a genome-wide significance threshold set at P<7.31×10^-6^. Manhattan plots and volcano plots were generated using the ggplot2 package to visualize results.

### Causal inference and colocalization analysis

Summary-data-based Mendelian Randomization (SMR) software (version 1.3.1) was employed to evaluate causal relationships between gene expression and psoriasis. SMR analysis utilizes eQTLs as instrumental variables, combined with Heterogeneity in Dependent Instruments (HEIDI) test to distinguish causal associations from spurious associations due to linkage disequilibrium. A HEIDI P>0.01 threshold was set for passing the test, indicating that association signals were more likely driven by a single causal variant. For genes passing the SMR-HEIDI test, Bayesian colocalization analysis was performed using the coloc package (version 5.1.0) to assess whether eQTL and GWAS signals shared the same causal variant. PPH4>0.8 was set as the threshold for strong support of colocalization.

### Subject recruitment and ethical approval

This study were approved by the Clinical Research Ethics Committee of General Hospital of Fushun Mining Bureau of Liaoning Health Industry Group (Approval number: 20240425). For long-read sequencing studies, 15 healthy controls (8 males, 7 females, aged 25–45 years) and 10 psoriasis patients (6 males, 4 females, aged 28–50 years, PASI scores 8-25) were recruited. For single-cell sequencing studies, 4 moderate-to-severe psoriasis patients (PASI>10) were enrolled. All participants provided written informed consent. Exclusion criteria included: pregnancy, lactation, systemic immunosuppressive therapy within 4 weeks prior to enrollment, concurrent autoimmune diseases, and acute infections.

### Sample collection and processing

Peripheral blood mononuclear cell (PBMC) isolation: 10mL of peripheral venous blood was collected in EDTA anticoagulant tubes and processed within 4 hours. PBMCs were isolated using Ficoll-Paque PLUS (GE Healthcare) density gradient centrifugation: whole blood was diluted 1:1 with PBS, carefully layered onto Ficoll medium, and centrifuged at 400×g for 30 minutes (brake off). The PBMC layer was collected, washed twice with PBS, counted, and immediately lysed in TRIzol reagent (Invitrogen) for storage at -80 °C.

Skin tissue processing: 4mm skin punch biopsies were obtained under local anesthesia from lesional and non-lesional skin (>5cm from lesion edge). Tissues were immediately placed in RPMI 1640 medium containing antibiotics. After mincing, tissues were digested in solution containing 2mg/mL collagenase IV (Worthington), 0.1mg/mL DNase I (Sigma), and 1% BSA at 37 °C with shaking for 45 minutes. Gentle pipetting was performed every 15 minutes to facilitate digestion. The suspension was filtered through 70μm cell strainers, and viable cells were enriched using dead cell removal kit (Miltenyi Biotec) magnetic bead selection, achieving >85% viability.

### Third-generation long-read sequencing

#### RNA extraction and quality control

Total RNA was extracted using TRIzol-chloroform method, followed by DNase I (Invitrogen) treatment to remove genomic DNA contamination. RNA concentration and purity were assessed using NanoDrop 2000 (OD260/280>1.8), and RNA integrity was evaluated using Agilent 2100 Bioanalyzer (RIN>7).

#### Library construction and sequencing

Libraries were constructed using Oxford Nanopore Technologies Direct RNA Sequencing Kit (SQK-RNA002). 500ng polyA+ RNA was processed following standard protocols for adapter ligation. Sequencing was performed on MinION sequencer using R9.4.1 flow cells, with 48-hour runs per sample to obtain >1M reads.

#### Data analysis

Base calling was performed using Guppy (v5.0.11), and reads were aligned to the human reference genome GRCh38 using minimap2 (v2.17). Alternative splicing events were identified and quantified using FLAIR (v1.5), calculating percent spliced-in (PSI) values. Alternative polyadenylation was analyzed using the TAPIS pipeline, calculating Proximal-Distal Index (PDI) values by comparing usage ratios of proximal versus distal poly(A) sites. Between-group differences were assessed using Wilcoxon rank-sum test.

#### Genome browser visualization

To visualize long-read sequencing coverage at the TDRKH 3′UTR locus, aligned BAM files from control and psoriasis samples were merged by group using samtools (v1.15). Per-base coverage was calculated using samtools depth across the TDRKH 3′UTR region (chr1:151,766,200–151,774,800, GRCh38). Coverage tracks were plotted using the Gviz package (v1.42) in R, with separate panels for control (n = 15) and psoriasis (n = 10) samples. Polyadenylation site positions were annotated based on PolyA_DB v3 database and validated by visual inspection of read termination patterns. Gene structure annotations were derived from GENCODE v38.

### 3’UTR reporter gene assays

#### Vector construction

TDRKH short 3’UTR (using proximal poly(A) site, 450bp) and long 3’UTR (using distal poly(A) site, 1,200bp) sequences were PCR-amplified from healthy donor PBMC cDNA. Amplified products were cloned into pcDNA3.1-βglobin vector using In-Fusion HD Cloning Kit (Takara), replacing the original 3’UTR. All constructs were verified by Sanger sequencing.

#### Cell transfection and mRNA stability detection

HEK293T cells were cultured in DMEM supplemented with 10% FBS. 2μg plasmids were transfected using Lipofectamine 3000 at a 3:1 ratio. 48 hours post-transfection, 5μg/mL actinomycin D was added to inhibit transcription, and cells were collected at 0, 2, 4, 6, 8, and 12 hours. RNA was extracted using TRIzol, reverse transcribed, and β-globin mRNA levels were detected using SYBR Green qPCR with GAPDH as internal control. Four independent biological replicates were performed. mRNA half-lives were calculated by fitting first-order decay curves using GraphPad Prism nonlinear regression.

### Single-Cell RNA sequencing

#### Library construction

Single-cell suspensions were adjusted to 1,000 cells/μL and captured using 10x Genomics Chromium Controller. Libraries were constructed following Chromium Single Cell 3’ v3.1 kit protocols: gel beads-in-emulsion (GEM) generation, reverse transcription, cDNA amplification, fragmentation, and adapter ligation. Library quality was assessed using Qubit and Bioanalyzer.

#### Sequencing and data processing

Paired-end sequencing (28bp+91bp) was performed on Illumina NovaSeq 6000 platform, targeting 50,000 reads per cell. Data processing was performed using Cell Ranger (v6.0.0): cellranger mkfastq for sample demultiplexing, cellranger count for alignment and gene expression quantification.

#### Data analysis

Downstream analysis was performed using Scanpy (v1.9) in Python. Quality control criteria included: 200 < number of detected genes < 6,000, total UMI count > 500, and mitochondrial gene percentage < 20% (**Supplementary Figure 1**). Data from all four patients were concatenated into a single dataset and normalized using scran size factors with log1p transformation. Highly variable genes (HVGs) were identified using the Seurat v3 method (top 3,000 genes). To prevent circularity in downstream analyses, TDRKH was explicitly excluded from the HVG set prior to dimensionality reduction. Principal component analysis was performed using the top 50 components at the global level. Patient-specific batch effects were corrected using the Harmony algorithm (24), with patient identity as the batch key (max iterations = 20) (**Supplementary Figures 2**, **S3**). UMAP embedding was computed using n_neighbors = 30 and min_dist = 0.3. Cell type annotation was performed using Leiden clustering (resolution 0.5) and validated against canonical lineage markers (**Supplementary Figure 4**), identifying 17 cell types across 17,734 cells. For macrophage subpopulation analysis, 954 macrophages/monocytes were re-extracted and re-clustered with TDRKH again excluded from feature selection. PCA was performed using 30 components, with n_neighbors = 20 and Leiden resolution = 0.8. Ten macrophage/myeloid subpopulations were identified and annotated based on established marker genes with literature references (**Supplementary Table 1**). To validate the robustness of this approach, we compared clustering results with and without TDRKH in the HVG set, obtaining Adjusted Rand Index = 0.979 and Normalized Mutual Information = 0.982 (**Supplementary Figure 7**), confirming that TDRKH exclusion did not alter the clustering structure.

#### TDRKH expression stratification and pathway scoring

For the TDRKH-high vs. TDRKH-low analysis, macrophages were stratified based on TDRKH expression using a median split: cells with TDRKH expression above the median of all TDRKH-expressing cells (log-normalized expression > 0) were classified as TDRKH-high (n = 429), and the remainder as TDRKH-low (n = 525). Pathway activation scores were calculated using the sc.tl.score_genes function in Scanpy, which computes the mean expression of pathway gene sets minus the mean expression of a reference set of randomly selected control genes matched for expression level. Three pathway gene sets were defined based on literature: (1) IL-23/IL-17 axis (macrophage-derived): IL23A, IL12A, IL12B, IL1B, CCL20, CXCL1, CXCL2, CXCL3; (2) TNF-α/NF-κB signaling: TNF, NFKB1, NFKB2, RELA, RELB, IKBKB, TNFAIP3, BCL2A1; (3) Oxidative damage response: SOD2, NOS2, HMOX1, NQO1, GCLM, TXNRD1. Gene sets were curated to include only genes with documented expression in myeloid cells. Differences in pathway scores between TDRKH-high and TDRKH-low groups were assessed using one-sided Mann-Whitney U test. Heatmap visualization of inflammatory gene expression was generated using row-wise Z-score normalization of log-normalized expression values, plotted with the pheatmap package in R.

### Cell culture and functional experiments

#### THP-1 cell differentiation and pro-inflammatory polarization

THP-1 cells (ATCC TIB-202) were cultured in RPMI 1640 medium containing 10% FBS, 100 U/mL penicillin, and 100 μg/mL streptomycin. The pro-inflammatory polarization protocol was as follows: (1) 100 ng/mL PMA treatment for 48 hours to induce monocyte-to-macrophage differentiation; (2) fresh medium replacement and 24-hour rest; (3) 100 ng/mL LPS + 20 ng/mL IFN-γ treatment for 24 hours to induce pro-inflammatory activation. Flow cytometry detection of CD80, CD86, and HLA-DR confirmed the pro-inflammatory phenotype.

#### siRNA transfection

Three siRNAs targeting TDRKH were designed (target sequences: siRNA1: 5′-GCAUGAACUUGCUGAAGAA-3′; siRNA2: 5′-GGACUUAGAUGCUGAAGUA-3′; siRNA3: 5′-GCAGGAAUUUGAAGAAGCA-3′). Reverse transfection was performed using Lipofectamine RNAiMAX: 9μL RNAiMAX + 150μL Opti-MEM per well in 6-well plates, mixed with 50nM siRNA, incubated at room temperature for 20 minutes. Cell suspension was added to a final volume of 2mL. Knockdown efficiency was verified by RT-qPCR 48 hours post-transfection using TDRKH-specific primers (forward: 5′-ATGGCAGCTTCAGATCTGG-3′; reverse: 5′-TCAGGTCACATGCTCAATGG-3′) with GAPDH as internal reference (forward: 5′-GAAGGTGAAGGTCGGAGTC-3′; reverse: 5′-GAAGATGGTGATGGGATTTC-3′).

Flow cytometry for surface marker analysis: Following siRNA transfection and LPS + IFN-γ stimulation, THP-1 macrophages were harvested and stained for surface markers. Cells were first incubated with Fc receptor blocking reagent (BioLegend) for 10 minutes at 4 °C, then stained with anti-CD80-FITC, anti-CD86-PE, and anti-HLA-DR-APC antibodies (all from BioLegend) for 30 minutes at 4 °C in the dark. Live/Dead Fixable Aqua Dead Cell Stain (Invitrogen) was used to exclude dead cells. Cells were acquired on a BD FACSCanto II flow cytometer (10,000 events per sample), and data were analyzed using FlowJo v10. Mean fluorescence intensity (MFI) was compared between siCtrl and siTDRKH groups using Student’s t-test (n = 3 biological replicates).

#### Inflammatory gene expression analysis

Total RNA was extracted from siCtrl and siTDRKH THP-1 macrophages (with and without LPS + IFN-γ stimulation) using TRIzol reagent. Reverse transcription was performed using PrimeScript RT Master Mix (Takara). Expression of inflammatory cytokines (IL6, TNF, IL1B, IL23A, IL12A) was quantified by SYBR Green qPCR using gene-specific primers (**Supplementary Table 2**), with GAPDH as internal control. Relative expression was calculated using the 2^-DDct^ method. Between-group comparisons were performed using Welch’s t-test (n = 3 biological replicates per condition). Results are presented as a row-normalized Z-score heatmap of log_2_-transformed relative expression values across four conditions (Ctrl, siTDRKH, Ctrl + LPS + IFN-γ, siTDRKH + LPS + IFN-γ).

### piRNA sequencing and analysis

Small RNA library construction: Total RNA was extracted using TRIzol, and 18-40nt small RNAs were separated using 15% urea-polyacrylamide gel electrophoresis. Target fragments were gel-excised and recovered. Libraries were constructed using NEBNext Multiplex Small RNA Library Prep Set: 3’ adapter ligation with T4 RNA ligase 2, 5’ adapter ligation with T4 RNA ligase 1, reverse transcription with SuperScript III, PCR amplification for 12 cycles, and 140-160bp product purification on 6% polyacrylamide gel.

Sequencing and bioinformatics analysis: Single-end 50bp sequencing was performed on Illumina HiSeq 2500 platform, >20M reads per sample. Adapters were removed using cutadapt, and quality control was performed using FastQC. Alignment to hg38 was performed using Bowtie (v1.2.3) allowing 1 mismatch, with unmapped reads further aligned to piRBase database. Reads of 26-32nt with 5’ U preference were defined as piRNAs. Differential expression analysis was performed using DESeq2, with |log2FC|>1 and P<0.05 considered significant. Target genes were predicted using miRanda (free energy <-20 kcal/mol, score >140), focusing on target sites in promoter regions (2kb upstream of TSS), 5’UTR, and 3’UTR.

### Statistical analysis

All statistical analyses were performed using R (v4.2.0). Normally distributed data were presented as mean ± standard error, with between-group comparisons using Student’s t-test; non-normally distributed data were presented as median (interquartile range), using Wilcoxon rank-sum test. Multiple group comparisons employed one-way ANOVA or Kruskal-Wallis test. Correlation analyses used Pearson or Spearman correlation coefficients. Multiple testing correction employed Bonferroni or Benjamini-Hochberg methods. Data visualization was performed using ggplot2, pheatmap, and other packages. All experiments were performed with at least 3 biological replicates, with P<0.05 considered statistically significant.

## Results

### Identification of psoriasis-associated genes through transcriptome-wide association analysis

**Figure 1:**
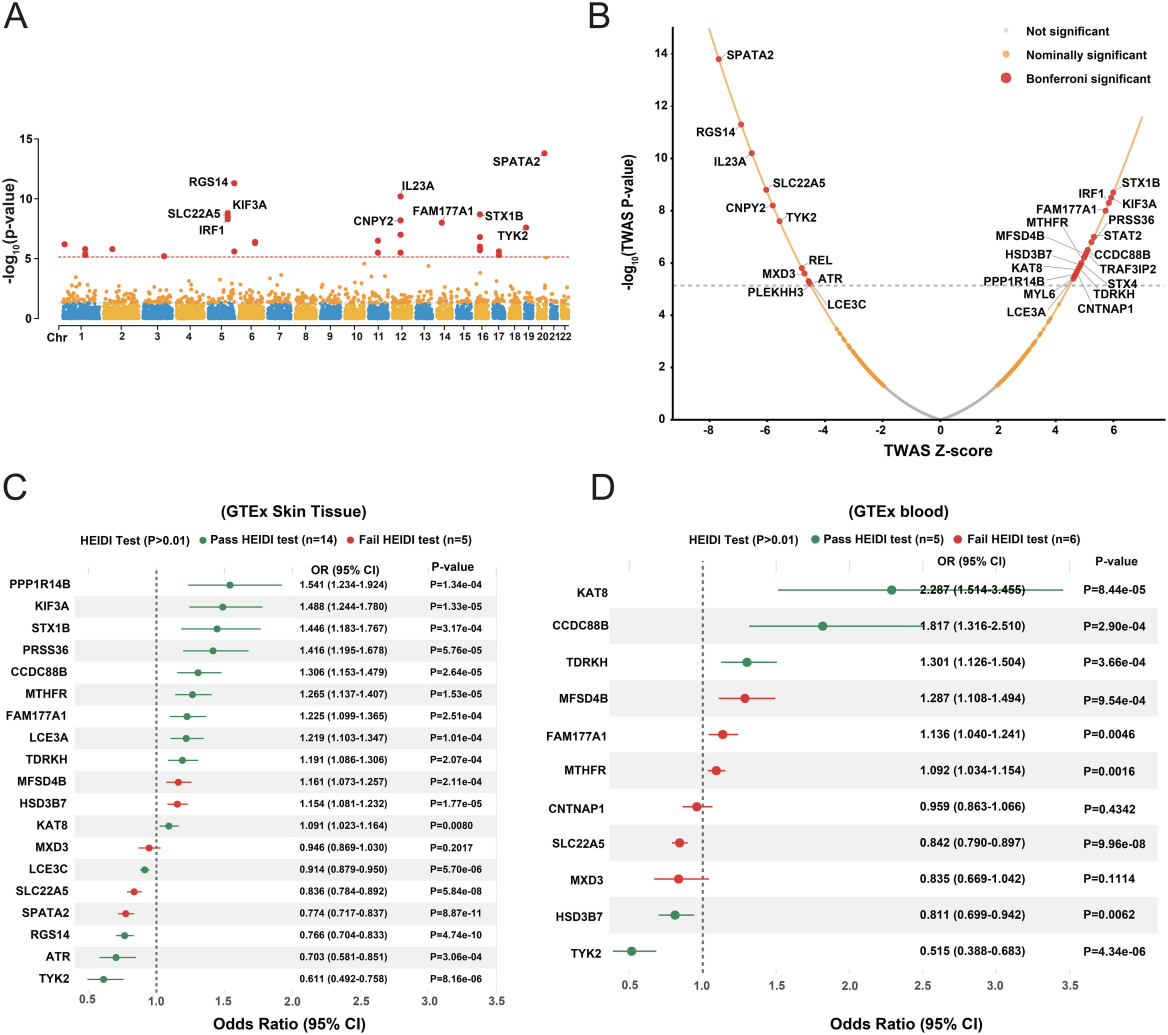
Transcriptome-wide association study identifies psoriasis susceptibility genes. **(A)** Manhattan plot of TWAS results showing the association between genetically predicted gene expression and psoriasis risk across the genome. Blue dots represent nominally significant genes (P < 0.05), orange dots indicate Bonferroni-significant genes (P < 7.31 × 10^-6^). The y-axis shows -log_10_(P-value) and x-axis shows chromosomal positions. Dashed horizontal line indicates the Bonferroni significance threshold. **(B)** Volcano plot displaying TWAS results with effect size (TWAS Z-score) on the x-axis and -log_10_(P-value) on the y-axis. Red dots represent Bonferroni-significant genes, orange dots indicate nominally significant genes, and gray dots show non-significant genes. Gene names are labeled for selected significant loci. Dashed horizontal line marks the Bonferroni significance threshold. **(C)** Forest plot showing SMR results for psoriasis-associated genes in GTEx skin tissue (non-sun-exposed). Green dots indicate genes passing the HEIDI test (P > 0.01), suggesting single causal variants, while red dots represent genes failing the HEIDI test (P ≤ 0.01), indicating potential linkage. Horizontal lines show 95% confidence intervals for odds ratios. P-values from SMR analysis are displayed on the right. **(D)** Forest plot displaying SMR results for psoriasis-associated genes in GTEx blood tissue. Color coding and interpretation are the same as in **(C)** Odds ratios with 95% confidence intervals and corresponding P-values are shown for each gene.

By applying the FUSION method for TWAS, we systematically evaluated psoriasis-associated expression quantitative trait loci in non-sun-exposed skin tissues. The genome-wide TWAS analysis identified 29 significantly associated gene loci under Bonferroni-corrected threshold (P = 7.31×10^-6^) (**Figure 1A**). These loci demonstrated scattered distribution across multiple chromosomes, with chromosome 6 harboring the most prominent signals, including SPATA2 (-log10 P-value > 14), RGS14, and IL23A (-log10 P-value > 10) (**Figure 1B**). Among the 29 significantly associated genes, multiple known immune-related genes (IL23A, STAT2, IRF1, KIF3A) and previously reported psoriasis susceptibility genes (CNPY2, SLC22A5, TYK2) were identified.

To validate the causality of TWAS findings, we performed in-depth analysis using SMR combined with HEIDI test. Within GTEx skin tissue data, 19 genes demonstrated significant evidence of causal associations, with 14 passing the HEIDI test (P > 0.01), indicating these associations were more likely driven by single causal variants (**Figure 1C**). PPP1R14B exhibited the strongest evidence of causal association (OR = 1.541, 95% CI: 1.234-1.924, P = 1.34×10^-4^), followed by KIF3A and STX1B. Notably, although SPATA2 showed the strongest signal in TWAS analysis, its SMR effect size was relatively modest and failed the HEIDI test, suggesting multiple independent causal variants at this locus.

**Figure 2:**
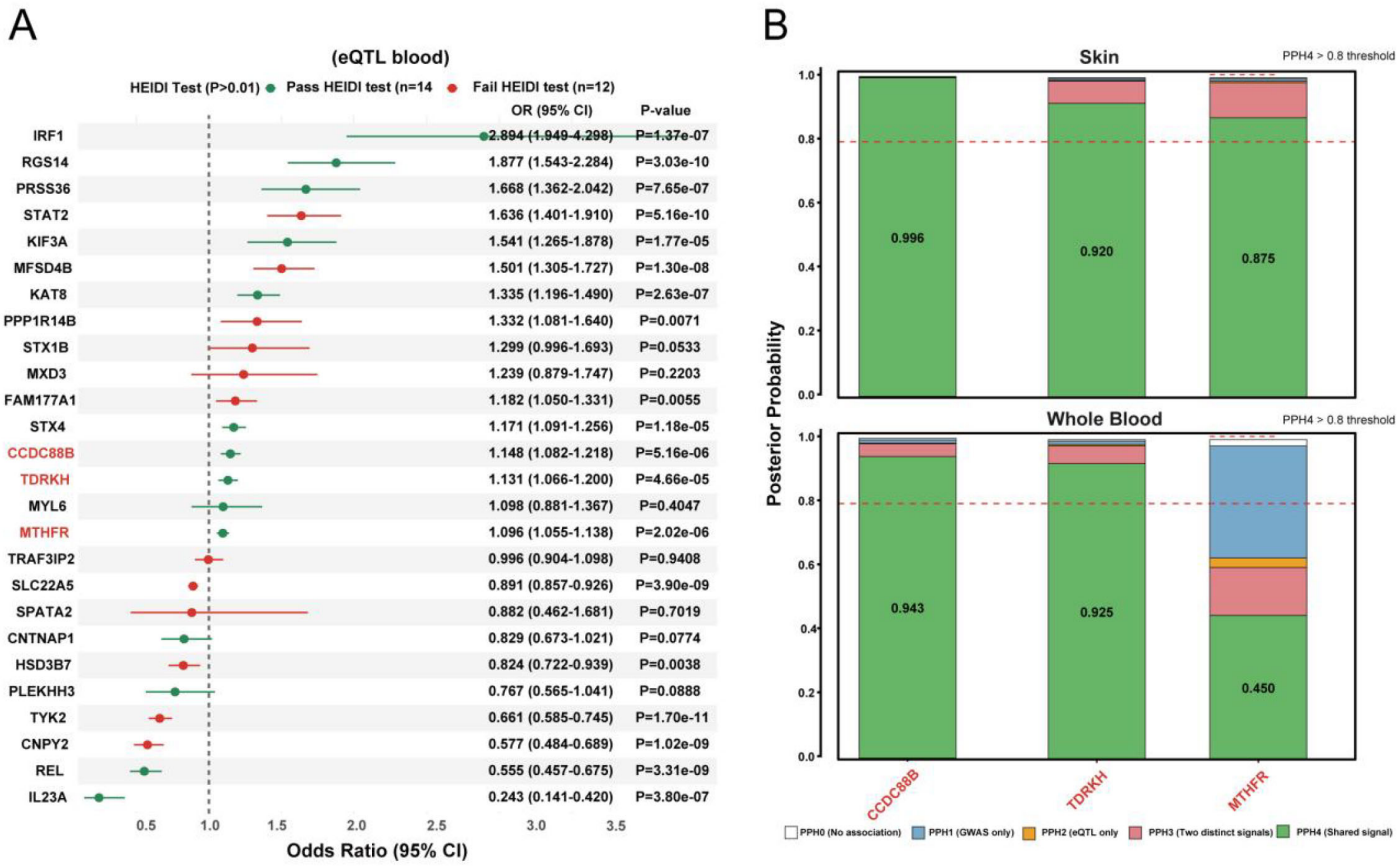
Cross-tissue validation and colocalization analysis of psoriasis susceptibility genes. **(A)** Forest plot showing SMR results for psoriasis-associated genes using the eQTLGen blood dataset (n=31,684). Green dots indicate genes passing the HEIDI test (P > 0.01), suggesting single causal variants, while red dots represent genes failing the HEIDI test (P ≤ 0.01), indicating potential linkage. The three core genes identified across all analyses (CCDC88B, TDRKH, and MTHFR) are highlighted in red text. Horizontal lines show 95% confidence intervals for odds ratios, with P-values displayed on the right. **(B)** Bayesian colocalization analysis results for the three core genes (CCDC88B, TDRKH, and MTHFR) in skin (upper panel) and whole blood (lower panel) tissues. Stacked bar plots show the posterior probabilities for five hypotheses: PPH0 (no association), PPH1 (GWAS only), PPH2 (eQTL only), PPH3 (two distinct signals), and PPH4 (shared signal). Values within bars indicate PPH4 probabilities. The dashed horizontal line marks the PPH4 > 0.8 threshold for strong evidence of colocalization.

Subsequently, we conducted cross-tissue validation in GTEx blood tissue. Blood tissue analysis identified 11 associated genes, with 5 passing the HEIDI test (**Figure 1D**). Particularly, CCDC88B, TDRKH, and MTHFR showed consistent associations across both tissues, while TYK2 demonstrated protective effects in blood (OR = 0.515, 95% CI: 0.388-0.683, P = 4.34×10^-6^). To further enhance statistical power, we utilized the eQTLGen blood dataset comprising 31,684 samples for large-scale validation. Among 26 detected associated genes, 14 passed the HEIDI test, with IRF1 showing the strongest effect (OR = 2.894, 95% CI: 1.949-4.298, P = 1.37×10^-7^) (**Figure 2A**).

Integrating results from multiple datasets, we ultimately identified three core genes showing robust associations across all validations: CCDC88B, TDRKH, and MTHFR. Bayesian colocalization analysis further supported these findings (**Figure 2B**). In skin tissue, all three genes demonstrated extremely high colocalization probability (PPH4 > 0.875), particularly CCDC88B reaching 0.996. In whole blood tissue, CCDC88B and TDRKH maintained high colocalization (PPH4 > 0.92), while MTHFR presented a more complex genetic architecture.

### Post-transcriptional regulatory landscape of candidate genes revealed by long-read sequencing

To further investigate the functional mechanisms of TWAS-identified genes in psoriasis, we employed third-generation long-read sequencing technology to analyze full-length transcripts from PBMCs of 15 healthy controls and 10 psoriasis patients, focusing on post-transcriptional regulatory features of the 29 candidate genes.

**Figure 3:**
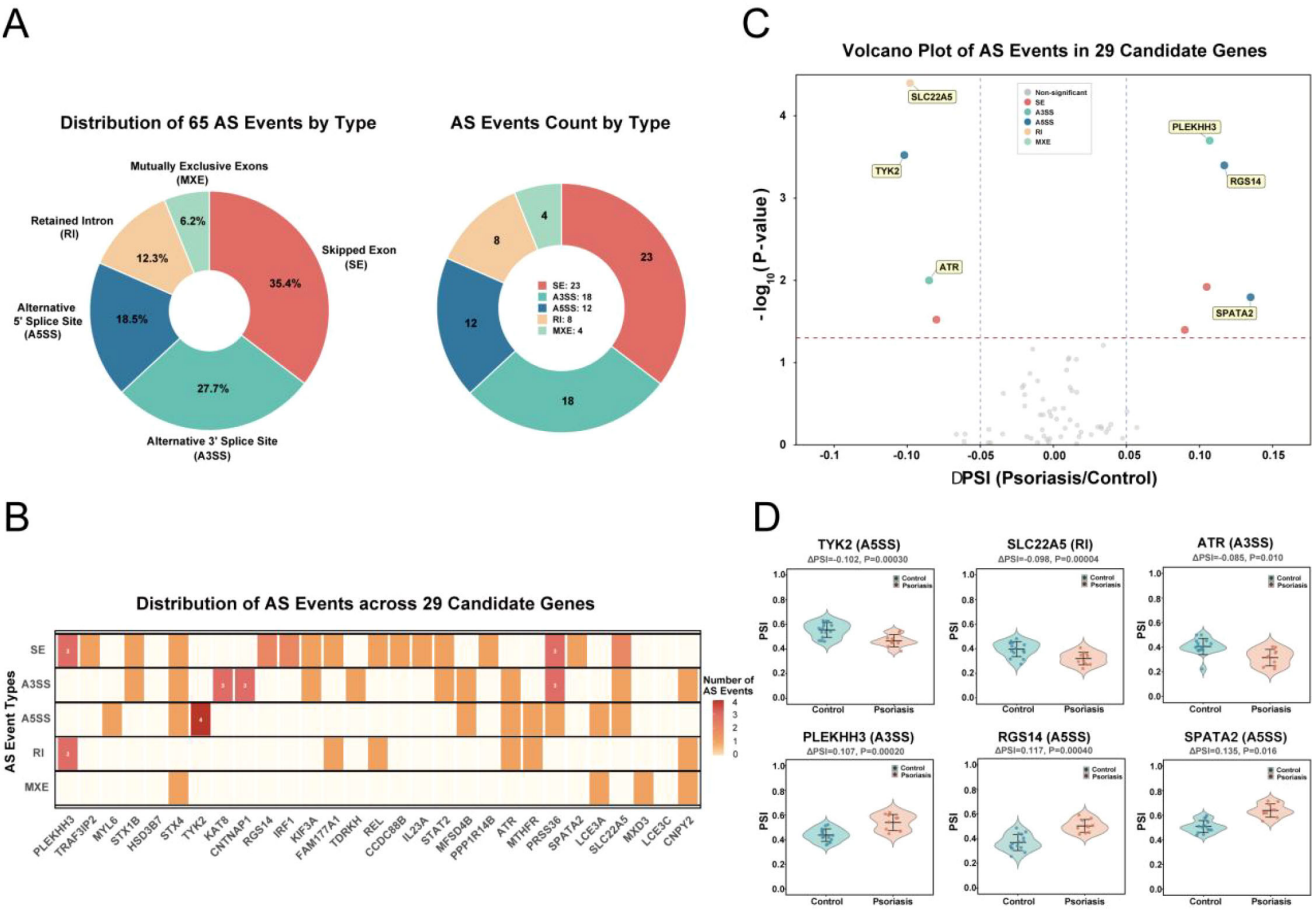
Alternative splicing landscape and differential splicing events of psoriasis candidate genes. **(A)** Distribution of 65 detected AS events across five categories. Donut charts show the proportion and count of each type, with SE being the most frequent. **(B)** Heatmap showing the distribution of AS events across 29 candidate genes. Color intensity and numbers indicate the count of events per gene, highlighting the splicing complexity of genes such as TYK2 and STX4. **(C)** Volcano plot displaying differential AS events between psoriasis patients and controls. The x-axis represents the change in Percent Spliced In (delta PSI) and the y-axis shows -log_10_(P-value). Significant events (P < 0.05) are colored by AS type and labeled with gene names. **(D)** Violin plots of six representative differential AS events. Each panel compares PSI values between Control (blue) and Psoriasis (orange) groups, with individual data points overlaid. Specific delta PSI and P-values are indicated above each plot. Data are presented as mean ± SEM.

A total of 65 AS events were detected across the 29 candidate genes, with distinct type distribution preferences (**Figure 3A**). Skipped exon (SE) events predominated (35.4%, 23 events), followed by alternative 3’ splice sites (A3SS, 27.7%) and alternative 5’ splice sites (A5SS, 18.5%), while intron retention (RI, 12.3%) and mutually exclusive exons (MXE, 6.2%) were relatively rare. This distribution pattern indicates that exon skipping and splice site selection represent the primary modes of splicing regulation.

AS event distribution showed significant heterogeneity among different genes (**Figure 3B**). TYK2 and STX4 exhibited the highest AS event density (4 events each), PLEKHH3, TRAF3IP2, RGS14, SPATA2, and LCE3A also demonstrated high splicing complexity (3 events each). In contrast, approximately one-third of candidate genes showed no detectable AS events, including the previously identified core genes CCDC88B and MTHFR, with only TDRKH displaying moderate splicing activity.

Comparing splicing patterns between patients and controls revealed that 9 of 65 AS events showed significant differences (P < 0.05) across 6 genes (**Figure 3C**). TYK2 and SLC22A5 splicing events demonstrated the most significant differences (-log10 P-value > 4). Further analysis revealed differential directional changes: TYK2 A5SS and SLC22A5 RI events showed significantly decreased PSI values in patients (P = 0.0003 and P = 0.00004), splice site selection events in PLEKHH3, RGS14, and SPATA2 exhibited increased PSI values in patients (P < 0.05) (**Figure 3D**).

**Figure 4:**
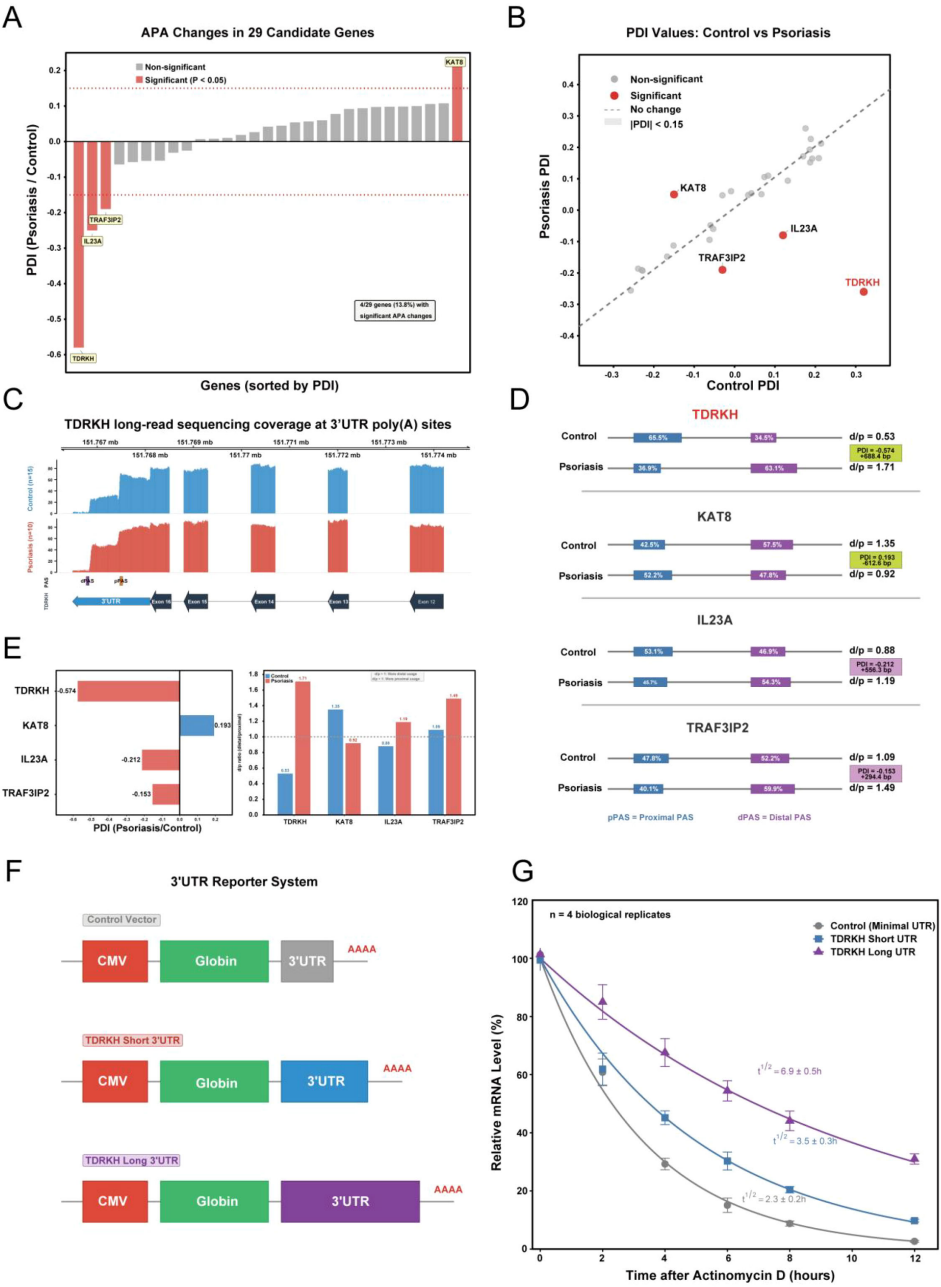
Alternative polyadenylation analysis reveals TDRKH as a key post-transcriptionally regulated gene in psoriasis. **(A)** Waterfall plot showing PDI values for 29 candidate genes in psoriasis patients. Bars are colored by significance: red indicates P < 0.05, gray indicates non-significant changes. Negative PDI values indicate preferential usage of the distal poly **(A)** site in psoriasis relative to controls. The four genes with significant APA changes (TDRKH, IL23A, TRAF3IP2, KAT8) are labeled. **(B)** Scatter plot comparing PDI values between controls (x-axis) and psoriasis patients (y-axis). Red dots indicate genes with significant APA changes (P < 0.05); gray dots show non-significant genes. The diagonal dashed line represents no change. TDRKH exhibits the most pronounced deviation from the diagonal. **(C)** Genome browser tracks showing long-read sequencing coverage at the TDRKH 3′UTR locus (chr1:151,766,200–151,774,800). Upper track: Control samples (n = 15, blue); lower track: Psoriasis samples (n = 10, red). The positions of the dPAS, distal PAS and pPAS, proximal PAS are annotated below the coverage tracks. Gene structure of TDRKH (Exons 12–16 and 3′UTR) is shown at the bottom. **(D)** Schematic representation of pPAS, proximal and dPAS, distal polyadenylation site usage in the four genes with significant APA changes. Blue bars represent proximal PAS usage percentage; purple bars show distal PAS usage. The distal-to-proximal (d/p) ratio and PDI values are shown for each gene in both controls and psoriasis patients. **(E)** Left panel: Bar chart showing PDI values (Psoriasis/Control) for the four differentially regulated genes. Right panel: Paired bar graph comparing d/p ratios between controls (blue) and psoriasis patients (red). Dashed line at d/p = 1 separates proximal-dominant from distal-dominant usage. **(F)** Schematic diagram of the 3′UTR reporter system. Three constructs were generated: control vector with minimal 3′UTR, TDRKH short 3′UTR (proximal PAS), and TDRKH long 3′UTR (distal PAS). All constructs share a CMV promoter and Globin coding sequence. **(G)** mRNA decay curves following actinomycin D treatment in HEK293T cells. Relative mRNA levels (%) are plotted against time (hours). Gray circles: control minimal 3′UTR (t½ = 2.3 ± 0.2 h); blue squares: TDRKH short 3′UTR (t½ = 3.5 ± 0.3 h); purple triangles: TDRKH long 3′UTR (t½ = 6.9 ± 0.5 h). Error bars represent mean ± SEM from n = 4 biological replicates.

Beyond alternative splicing, we analyzed APA patterns across the 29 candidate genes. Results showed that 4 genes (13.8%) exhibited significant APA changes between patients and controls (**Figure 4A**). TDRKH demonstrated the most significant change, with a delta PDI of −0.574, reflecting a pronounced shift from proximal to distal poly(A) site usage and consequent 3′UTR lengthening. IL23A showed a similar lengthening trend (delta PDI = −0.212), while TRAF3IP2 (delta PDI = −0.153) and KAT8 (delta PDI = 0.193) also exhibited significant APA alterations.

Scatter plot analysis further confirmed this differential distribution pattern (**Figure 4B**). Most genes maintained stable PDI values between groups, clustering near the diagonal, while the 4 differential genes clearly deviated. Specific poly(A) site analysis revealed that TDRKH proximal poly(A) site usage decreased from 65.5% to 36.9% in patients (P < 0.001), with distal-to-proximal usage ratio (d/p ratio) increasing from 0.53 to 1.71 (**Figures 4D, E**). IL23A and TRAF3IP2 d/p ratios also increased in patients, indicating a general tendency toward distal poly(A) site usage in these genes during psoriasis.

Given that TDRKH demonstrated the most consistent evidence across multi-layered genetic analyses and was the only core gene showing both significant AS and APA alterations—with the most substantial APA changes among all candidate genes—we selected TDRKH as our primary research focus for subsequent studies.

To visualize the APA shift directly, we generated long-read sequencing coverage tracks at the TDRKH 3′UTR locus (**Figure 4C**). Control samples showed predominant read coverage terminating at the proximal PAS (pPAS), whereas psoriasis samples exhibited a clear shift toward the distal PAS (dPAS), with extended coverage across the additional 688 bp of 3′UTR sequence. The proximal-to-distal usage ratio shifted from pPAS-dominant (d/p = 0.53) in controls to dPAS-dominant (d/p = 1.71) in psoriasis (**Figures 4D, E**).

Based on this comprehensive evidence, we constructed reporter gene systems containing TDRKH short and long 3′UTRs to evaluate APA-mediated regulation of transcript stability in HEK293T cells (**Figure 4F**). mRNA stability analysis following actinomycin D treatment revealed that 3′UTR length significantly affected TDRKH transcript degradation rates (**Figure 4G**). TDRKH transcripts with short 3′UTRs exhibited faster degradation, with a half-life of 3.5 ± 0.3 hours, significantly shorter than the 6.9 ± 0.5 hours for long 3′UTR transcripts (P < 0.01). Control minimal 3′UTR constructs degraded most rapidly, with a half-life of only 2.3 ± 0.2 hours. At the 12-hour time point, short and long 3′UTR transcripts retained 10% and 30% of initial levels, respectively, demonstrating that 3′UTR lengthening significantly enhances mRNA stability. These results suggest that the shift from proximal to distal poly(A) sites in TDRKH observed in psoriasis patients may upregulate gene expression through increased mRNA stability.

### Cell-specific expression patterns of TDRKH in psoriatic lesional microenvironment

To comprehensively understand TDRKH expression patterns and cell specificity within the psoriatic lesional microenvironment, we performed single-cell RNA sequencing analysis on lesional and paired non-lesional skin tissues from 4 psoriasis patients.

**Figure 5:**
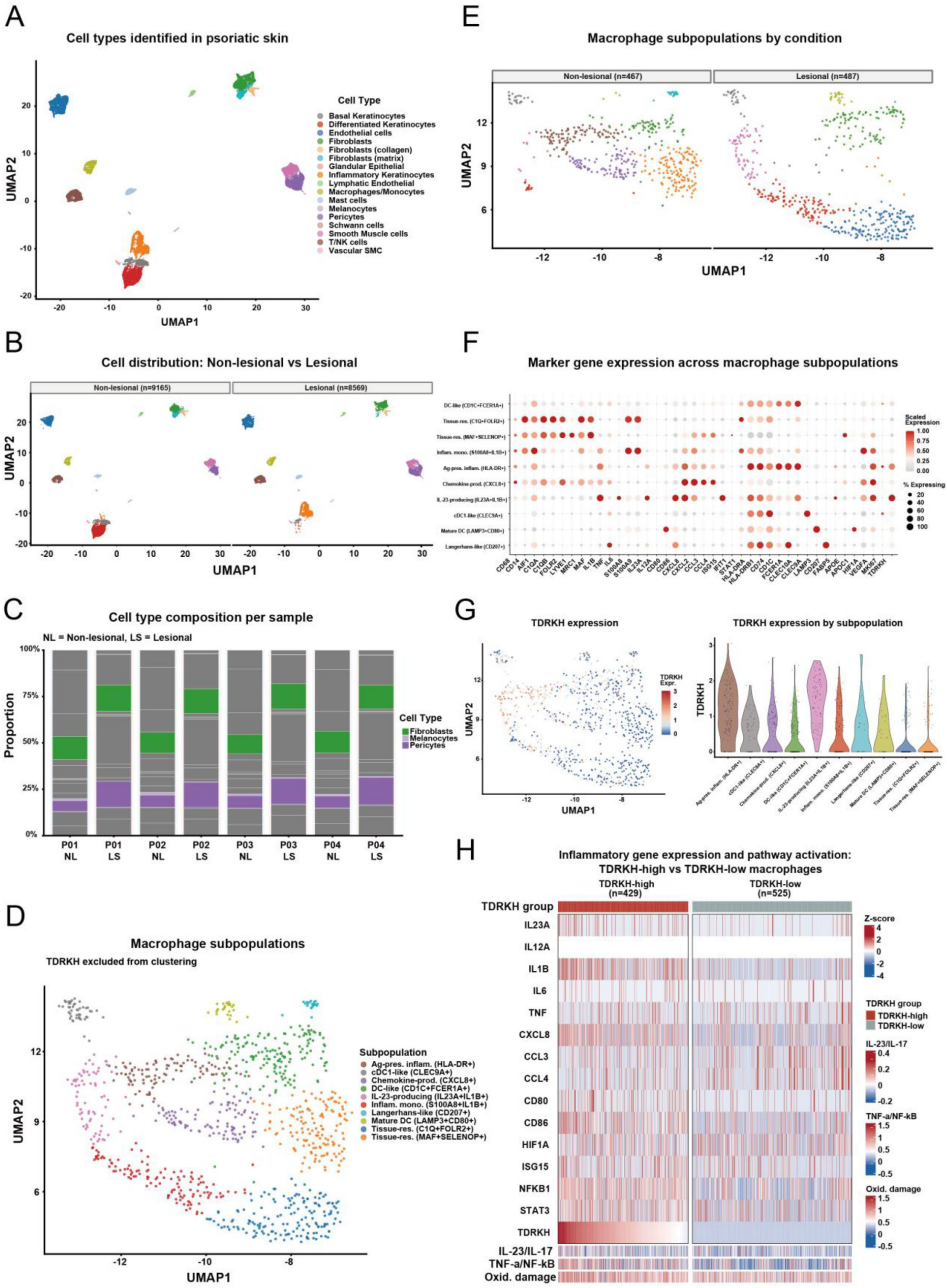
Single-cell RNA sequencing reveals TDRKH enrichment in pro-inflammatory macrophage subpopulations of psoriatic skin. **(A)** UMAP visualization of 17,734 cells from psoriatic lesional and paired non-lesional skin samples, identifying 17 major cell types. TDRKH was excluded from feature selection for dimensionality reduction and clustering. **(B)** Split UMAP comparing cell distribution between Non-lesional (n = 9,165) and Lesional (n = 8,569) conditions, showing expansion of inflammatory populations in lesional skin. **(C)** Stacked bar chart showing cell type composition for each sample across four patients (P01–P04). NL, non-lesional; LS, lesional. **(D)** Sub-clustering of 954 macrophages and monocytes into 10 functionally distinct subpopulations. Each cluster is labeled with its defining marker genes. **(E)** Split UMAP of macrophage subpopulations by condition (Non-lesional n = 467; Lesional n = 487), highlighting the enrichment of inflammatory subsets in lesional tissue. **(F)** Dot plot showing the expression of canonical marker genes across macrophage subpopulations. Dot size represents the percentage of cells expressing the gene; color intensity indicates the scaled mean expression level. TDRKH expression is shown at the bottom. **(G)** TDRKH expression patterns across macrophage subpopulations visualized by UMAP (left) and violin plot (right), demonstrating high expression in IL-23-producing and antigen-presenting clusters. **(H)** Heatmap and pathway enrichment analysis comparing TDRKH-high (n = 429) and TDRKH-low (n = 525) macrophages. The upper panel displays Z-score normalized expression of key inflammatory genes; the lower panel shows enrichment scores for IL-23/IL-17, TNF-a/NF-kB, and oxidative damage response pathways.

Following stringent quality control (**Supplementary Figure 1**) and Harmony batch correction across four patients (**Supplementary Figures 2**, **S3**), UMAP dimensionality reduction identified 17 cell types across 17,734 cells (**Figure 5A**). Cell type annotation was validated against canonical lineage markers (**Supplementary Figure 4**), identifying Basal Keratinocytes, Differentiated Keratinocytes, Inflammatory Keratinocytes, Fibroblasts (including collagen-producing and matrix-remodeling subtypes), Endothelial cells, Lymphatic Endothelial cells, Macrophages/Monocytes, T/NK cells, Mast cells, Melanocytes, Pericytes, Schwann cells, Smooth Muscle cells, Glandular Epithelial cells, and Vascular SMC. Notably, TDRKH was excluded from the highly variable gene set used for dimensionality reduction and clustering, ensuring that downstream TDRKH expression analyses are independent of the clustering procedure.

Split UMAP visualization comparing Non-lesional (n = 9,165 cells) and Lesional (n = 8,569 cells) samples revealed marked differences in cell type distribution (**Figure 5B**). Non-lesional skin was dominated by fibroblasts and differentiated keratinocytes, with few macrophages, while lesional skin exhibited substantial expansion of inflammatory keratinocytes and macrophages/monocytes. Stacked bar charts of cell type composition per sample (**Figure 5C**), with each patient’s lesional (LS) and non-lesional (NL) samples explicitly labeled, confirmed consistent macrophage enrichment in lesional tissue across all four patients (P01–P04).

Given the significant macrophage enrichment, we performed refined subpopulation analysis on 954 macrophages/monocytes. Re-clustering with TDRKH excluded from feature selection identified 10 functionally distinct subpopulations, each defined by established marker genes (**Figure 5D**; **Supplementary Table 1**): Tissue-resident macrophages (C1Q+FOLR2+ and MAF+SELENOP+), DC-like cells (CD1C+FCER1A+), Inflammatory monocytes (S100A8+IL1B+), Antigen-presenting inflammatory macrophages (HLA-DR+), Chemokine-producing macrophages (CXCL8+), IL-23-producing macrophages (IL23A+IL1B+), cDC1-like cells (CLEC9A+), Mature DCs (LAMP3+CD80+), and Langerhans-like cells (CD207+). Split UMAP visualization by condition (Non-lesional n = 467; Lesional n = 487) revealed selective enrichment of inflammatory monocytes and IL-23-producing macrophages in lesional tissues, while tissue-resident macrophages predominated in non-lesional skin (**Figure 5E**; **Supplementary Figure 5**). A comprehensive dot plot of 40+ marker genes validated the transcriptional identity of each subpopulation (**Figure 5F**).

Based on TDRKH’s consistent evidence from prior multi-layered analyses, we examined its expression across macrophage subpopulations. TDRKH showed highest expression levels in the IL-23-producing (IL23A+IL1B+) and Antigen-presenting inflammatory (HLA-DR+) subpopulations (**Figure 5G**). Stratifying macrophages into TDRKH-high (n = 429) and TDRKH-low (n = 525) groups revealed that TDRKH-high macrophages exhibited enhanced expression of pro-inflammatory genes, including IL23A, IL12A, IL1B, IL6, TNF, CXCL8, CCL3, CCL4, CD80, CD86, HIF1A, ISG15, NFKB1, and STAT3 (**Figure 5H**). Pathway enrichment analysis confirmed significant activation of the IL-23/IL-17 axis, TNF-α/NF-κB signaling, and oxidative damage response pathways in TDRKH-high macrophages (**Supplementary Figure 6**; all P < 0.01, Mann-Whitney U test). These results suggest that TDRKH participates in psoriasis pathogenesis through high expression in pro-inflammatory macrophage subsets, particularly the IL-23-producing subpopulation, promoting activation of key inflammatory pathways.

### TDRKH-Mediated piRNA regulation and functional validation in pro-inflammatory macrophages

**Figure 6:**
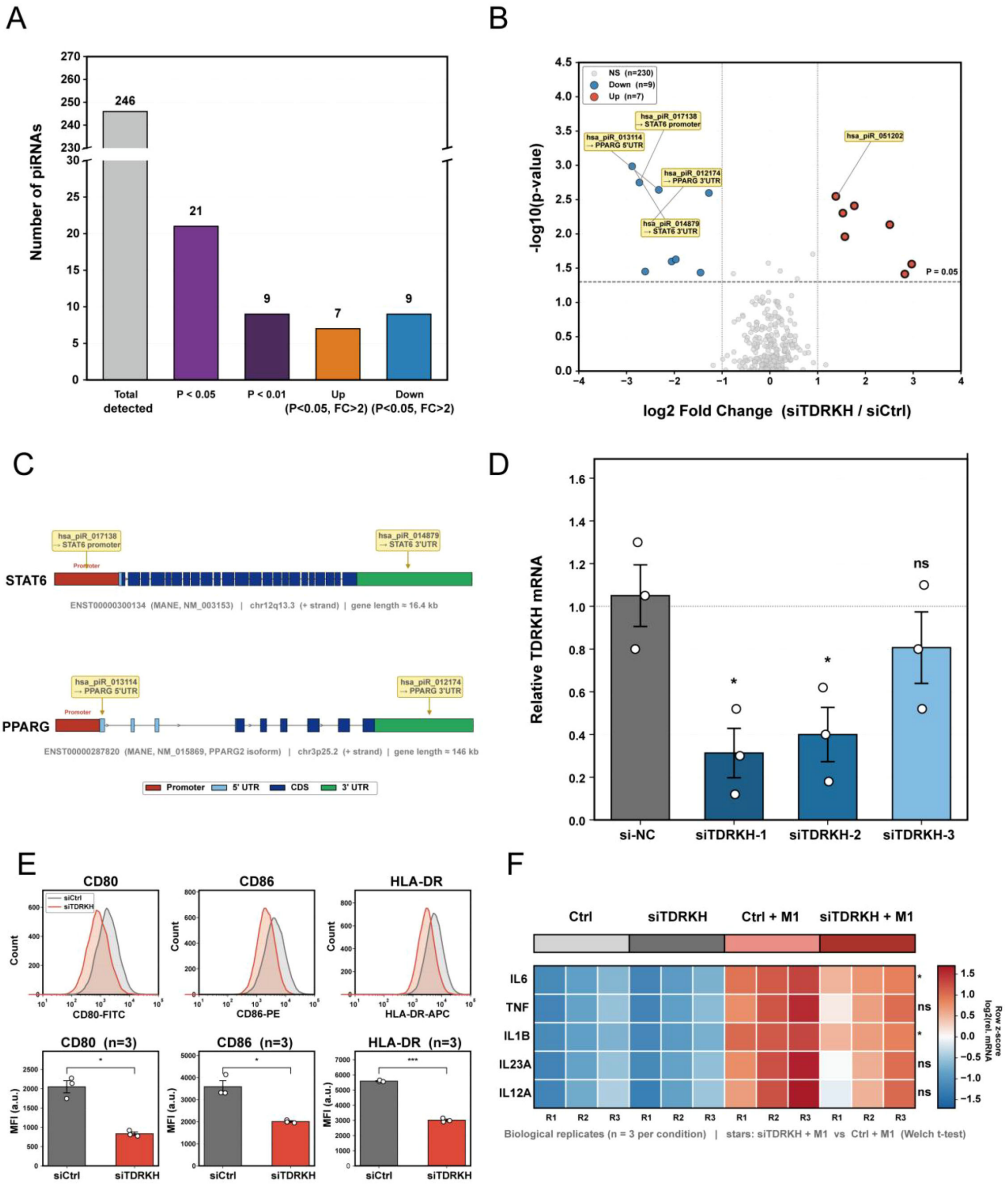
TDRKH knockdown alters piRNA expression and impairs pro-inflammatory phenotype in macrophages. **(A)** Bar graph summarizing piRNA differential expression analysis following TDRKH knockdown in LPS + IFN-γ–polarized THP-1 macrophages. Total detected piRNAs (gray, n = 246) and distribution across significance thresholds: P < 0.05 (purple, n = 21), P < 0.01 (dark purple, n = 9), upregulated with P < 0.05 and FC > 2 (orange, n = 7), and downregulated with P < 0.05 and FC > 2 (blue, n = 9). **(B)** Volcano plot displaying piRNA expression changes. The x-axis shows log_2_ fold change (siTDRKH/siCtrl); the y-axis shows −log_10_(P-value). Blue dots: significantly downregulated piRNAs (n = 9); red dots: significantly upregulated (n = 7); gray dots: non-significant (n = 230). Selected piRNAs with predicted immunoregulatory targets are annotated. **(C)** Schematic diagrams showing predicted targeting of immunoregulatory genes by differentially expressed piRNAs. Upper panel: STAT6 gene structure with hsa_piR_017138 targeting the promoter and hsa_piR_014879 targeting the 3′UTR. Lower panel: PPARG gene structure with hsa_piR_013114 targeting the 5′UTR and hsa_piR_012174 targeting the 3′UTR. Gene regions are color-coded: promoter (red), 5′UTR (light blue), CDS (dark blue), 3′UTR (green). **(D)** TDRKH knockdown efficiency measured by qPCR. Three independent siRNAs (siTDRKH-1, -2, -3) were tested against non-targeting control (si-NC). n = 3 biological replicates per condition. Individual data points are shown with bar heights representing mean ± SEM. *P < 0.05 vs. si-NC (Student’s t-test); ns, not significant. **(E)** Flow cytometry analysis of pro-inflammatory surface markers following TDRKH knockdown. Upper panels: representative histogram overlays of CD80-FITC, CD86-PE, and HLA-DR-APC fluorescence (gray: siCtrl; red: siTDRKH). Lower panels: quantification of MFI, mean fluorescence intensity from n = 3 biological replicates. *P < 0.05, ***P < 0.001 (Student’s t-test). **(F)** Heatmap of inflammatory cytokine expression across four conditions: Ctrl, siTDRKH, Ctrl + LPS + IFN-γ, and siTDRKH + LPS + IFN-γ. Row-normalized Z-scores of log_2_(relative mRNA) for IL6, TNF, IL1B, IL23A, and IL12A are shown. n = 3 biological replicates per condition. Significance annotations compare siTDRKH + LPS + IFN-γ vs. Ctrl + LPS + IFN-γ (Welch’s t-test): *P < 0.05; ns, not significant.

To further investigate TDRKH functional mechanisms in pro-inflammatory macrophages, we established an *in vitro* model using THP-1 cells polarized with LPS + IFN-γ. We first validated TDRKH knockdown efficiency using three independent siRNAs (**Figure 6D**). siTDRKH-1 and siTDRKH-2 achieved approximately 70% and 60% knockdown, respectively (P < 0.05), while siTDRKH-3 did not reach significance, serving as a specificity control. Subsequent functional experiments were performed using the two effective siRNAs.

TDRKH knockdown significantly altered the piRNA expression profile of LPS + IFN-γ–polarized THP-1 macrophages (**Figure 6A**). Among 246 detected piRNAs, 21 showed significant changes at P < 0.05 level, with 9 reaching P < 0.01. Applying more stringent statistical thresholds (P < 0.05, fold change > 2), 16 piRNAs (7 upregulated, 9 downregulated) maintained significant differences. Volcano plot analysis clearly illustrated this expression alteration pattern (**Figure 6B**). The most significantly upregulated piRNAs included hsa_piR_014628 (log2 FC = 3.2), hsa_piR_020362 (log2 FC = 3.1), and hsa_piR_021830 (log2 FC = 2.8), while the most prominently downregulated were hsa_piR_012174 (log2 FC = −2.5) and hsa_piR_017138 (log2 FC = −2.3). Notably, upregulated piRNAs generally showed greater fold changes than downregulated ones, suggesting TDRKH primarily functions as a negative regulator of certain piRNAs.

Target gene prediction analysis revealed the potential functional significance of these differential piRNAs (**Figure 6C**). Two pairs of significantly downregulated piRNAs were predicted to target key immunoregulatory genes: hsa_piR_017138 targeting the STAT6 promoter region and hsa_piR_014879 targeting the STAT6 3′UTR, while hsa_piR_013114 and hsa_piR_012174 were predicted to target PPARG 5′UTR and 3′UTR regions, respectively. STAT6 mediates IL-4/IL-13 signaling to promote anti-inflammatory macrophage programs, while PPARG exerts anti-inflammatory effects through NF-κB inhibition. These findings suggest that TDRKH may maintain the pro-inflammatory phenotype of macrophages by sustaining specific piRNA expression to suppress anti-inflammatory gene regulators.

To determine whether TDRKH knockdown functionally impairs the pro-inflammatory phenotype, we assessed surface marker expression by flow cytometry and inflammatory gene expression by qPCR (**Figures 6E, F**). TDRKH knockdown significantly reduced the surface expression of co-stimulatory molecules CD80 (P < 0.05) and CD86 (P < 0.05), as well as the antigen-presenting molecule HLA-DR (P < 0.001), compared to control THP-1 macrophages (**Figure 6E**). At the transcriptional level, qPCR analysis of five key inflammatory cytokines across four experimental conditions (Ctrl, siTDRKH, Ctrl + LPS + IFN-γ, siTDRKH + LPS + IFN-γ) revealed that TDRKH knockdown significantly attenuated IL6 (P < 0.05) and IL1B (P < 0.05) expression in LPS + IFN-γ–stimulated macrophages, while TNF, IL23A, and IL12A showed downward trends that did not reach statistical significance (**Figure 6F**). These data demonstrate that TDRKH knockdown impairs the pro-inflammatory phenotype of macrophages at both the surface marker and cytokine expression levels, providing direct functional evidence that the TDRKH–piRNA axis contributes to macrophage inflammatory activation.

## Discussion

This study systematically elucidated the genetic susceptibility mechanisms and post-transcriptional regulatory networks underlying psoriasis through integration of TWAS, SMR, and third-generation sequencing technologies. Our findings not only validated known psoriasis susceptibility genes but, more importantly, uncovered a novel mechanism whereby TDRKH participates in macrophage polarization through regulation of piRNA and alternative polyadenylation, providing new perspectives for understanding the molecular pathology of psoriasis.

Our TWAS analysis identified 29 significantly associated genes in skin tissue, corroborating recent findings from multiple studies. Tsoi et al.’s large-scale GWAS similarly reported significant associations with IL23A, TYK2, and other genes (25), while our expression quantitative trait loci analysis further confirmed the functional significance of these associations at the transcriptional level. Particularly noteworthy is that although SPATA2 showed the strongest signal in TWAS, it failed the HEIDI test, suggesting multiple independent causal variants at this locus, consistent with findings from the Genetic Investigation of ANthropometric Traits (GIANT) consortium in complex trait studies (26). SMR analysis identified three core genes (CCDC88B, TDRKH, MTHFR) demonstrating robust causal associations across multiple tissues. CCDC88B is preferentially expressed in immune cells and regulates leukocyte effector functions rather than acting in the Wnt pathway; aberrant Wnt signaling activation, in turn, has been demonstrated to play crucial roles in psoriatic keratinocyte proliferation (27, 28). The association between MTHFR C677T polymorphism and psoriasis susceptibility has been validated in multiple populations (29), with our findings further supporting the important role of folate metabolism in psoriasis pathogenesis.

Third-generation sequencing technology enabled comprehensive characterization of post-transcriptional regulatory features of candidate genes. The identification of 65 alternative splicing events revealed the complexity of gene expression regulation in psoriasis-associated genes, consistent with widespread splicing abnormalities reported by Schafer et al. in autoimmune diseases (30). The alterations in multiple TYK2 splice isoforms in patients merit particular attention, as TYK2 is a key signaling molecule in the IL-23/IL-17 axis (31), and different splice isoforms may possess varying signal transduction capabilities. Alternative polyadenylation analysis unveiled another crucial regulatory layer. The significant 3’UTR lengthening of TDRKH in patients, resulting in increased mRNA stability, carries important implications for inflammatory responses. While Mayr and Bartel’s seminal work demonstrated that 3’UTR shortening typically associates with cell proliferation (32), our observed 3’UTR lengthening may reflect specific regulatory requirements under chronic inflammatory conditions. Similar alterations in IL23A further support the important role of APA in regulating inflammatory gene expression.

The mechanism underlying the enhanced mRNA stability of the long 3′UTR isoform may involve specific cis-regulatory elements within the extended region. Analysis of publicly available eCLIP data from the ENCODE project and the RBPmap database identified several putative RNA-binding protein (RBP) recognition motifs in the region between the proximal and distal PAS of TDRKH, including binding sites for HuR (ELAVL1), a well-characterized mRNA stabilizer, and TTP (ZFP36), a destabilizing factor. The presence of HuR binding sites is particularly notable, as HuR-mediated stabilization through AU-rich elements in 3′UTRs is a well-established mechanism for post-transcriptional gene regulation in immune cells. The approximately 3-fold increase in mRNA half-life observed for the long 3′UTR isoform (t½ = 6.9 h vs. 2.3 h; **Figure 4G**) is consistent with HuR-mediated stabilization. However, experimental validation through RIP-qPCR or eCLIP would be needed to confirm direct RBP binding to the extended TDRKH 3′UTR.

The most significant finding of this study is the mechanism by which TDRKH regulates macrophage inflammatory activation through piRNA. TDRKH belongs to the Tudor domain protein family and participates in piRNA biogenesis in germline cells (33). However, its function in somatic immune regulation has rarely been reported. Our discovery that TDRKH is highly expressed in pro-inflammatory macrophage subsets, particularly the IL-23-producing (IL23A+IL1B+) subpopulation, and that its knockdown impairs the pro-inflammatory phenotype (reduced CD80, CD86, HLA-DR, IL6, and IL1B) through altered piRNA expression provides new evidence for understanding piRNA’s role in inflammatory diseases. TDRKH knockdown led to downregulation of piRNAs predicted to target STAT6 and PPARγ, both key transcription factors that promote anti-inflammatory macrophage programs (34, 35). Rather than viewing this through the lens of a binary M1/M2 switch, our single-cell data reveal that TDRKH-high macrophages exist along a spectrum of activation states, with enrichment in subpopulations driving the IL-23/IL-17 axis. This reveals that piRNAs may serve as fine-tuning regulators of macrophage activation, maintaining specific pro-inflammatory states through precise regulation of key transcription factor expression.

Single-cell RNA sequencing analysis not only confirmed massive macrophage infiltration in psoriatic lesions but also revealed their functional heterogeneity. The identification of ten macrophage/myeloid subpopulations, each defined by established marker genes and validated through robustness analysis (ARI = 0.979; **Supplementary Figure 7**), resonates with Reynolds et al.’s findings in atopic dermatitis (21), suggesting that inflammatory skin diseases may share certain macrophage activation patterns. The selective enrichment of IL-23-producing (IL23A+IL1B+) and inflammatory monocyte (S100A8+IL1B+) subpopulations in lesional tissue, together with high TDRKH expression in these subsets, provides direct evidence for their central role in disease pathogenesis. Particularly noteworthy is the significant activation of the IL-23/IL-17 axis in TDRKH-high macrophages. This finding organically links genetic susceptibility (TDRKH variants), post-transcriptional regulation (APA and piRNA), and functional phenotypes (IL-23 secretion), constructing a complete regulatory cascade from genotype to phenotype. This also explains why IL-23-targeting biologics demonstrate remarkable efficacy in psoriasis treatment (36).

Our findings carry important clinical translational value. First, TDRKH and its regulated piRNAs could serve as potential biomarkers for psoriasis. Second, targeting the TDRKH-piRNA axis may represent a novel therapeutic strategy. Considering that piRNAs can specifically regulate target genes through antisense mechanisms (13), designing antagonists against pro-inflammatory piRNAs could achieve precise immune modulation. Additionally, developing small molecule drugs that regulate APA processes warrants exploration (10).

Our findings complement and extend recent observations by Wang et al. (37), who employed integrative proteomics and transcriptomics analysis to identify TDRKH as a candidate therapeutic target for psoriasis with robust colocalization evidence (PPH4 > 0.9). Their single-cell analysis revealed TDRKH expression predominantly in T cells of psoriatic skin. Our study extends these findings by demonstrating that TDRKH is also highly expressed in pro-inflammatory macrophage subpopulations, particularly the IL-23-producing (IL23A+IL1B+) subset, and that it functionally regulates the macrophage inflammatory phenotype through piRNA-mediated mechanisms. Together, these studies suggest that TDRKH may exert cell type-specific roles in psoriasis pathogenesis, highlighting the emerging importance of the PIWI–piRNA pathway in somatic immune cells beyond its canonical germline functions.

This study has several limitations. First, while we validated TDRKH function at multiple levels, its upstream regulatory mechanisms remain unclear. Second, piRNA biogenesis and mechanisms of action in somatic cells may differ from those in germline cells, requiring further investigation. Finally, our functional experiments were primarily based on *in vitro* cell models, necessitating further validation in animal models and clinical samples. Future research should focus on: how TDRKH variants affect protein function and piRNA binding capacity; systematic elucidation of piRNA regulatory networks in macrophages; development of therapeutic strategies targeting the TDRKH-piRNA axis; and exploration of this regulatory mechanism in other inflammatory diseases.

## Conclusions

In conclusion, through multi-omics integrative analysis, this study revealed a novel mechanism whereby TDRKH, as a psoriasis susceptibility gene, influences macrophage function through post-transcriptional regulatory mechanisms. The crucial role of the TDRKH–piRNA axis in maintaining pro-inflammatory macrophage phenotypes provides new perspectives for understanding psoriasis molecular pathology and establishes theoretical foundations for developing novel therapeutic strategies.

## Data Availability

The data presented in the study are deposited in the National Genomics Data Center (NGDC), China National Center for Bioinformation/Beijing Institute of Genomics, Chinese Academy of Sciences, under BioProject accession number PRJCA063869.

## Glossary

APA: alternative polyadenylation
TWAS: Transcriptome-wide association study
GWAS: genome-wide association studies
eQTL: expression quantitative trait loci
SMR: summary-data-based Mendelian randomization
AS: Alternative splicing
piRNAs: PIWI-interacting RNAs
GTEx: Genotype-Tissue Expression
HEIDI: Heterogeneity in Dependent Instruments
PBMC: Peripheral blood mononuclear cell
PSI: percent spliced-in
PDI: proximal-Distal Index
HVGs: Highly variable genes
MFI: mean fluorescence intensity.
