# Supplementary material for: Post-transcriptional regulation via alternative polyadenylation and piRNA shapes macrophage responses in psoriasis
Source: Front Immunol. 2026 Jul 23;17:1760348. doi: 10.3389/fimmu.2026.1760348 (PMC13441757; doi:10.3389/fimmu.2026.1760348)

**Supplementary Figure S2: UMAP Overview (Harmony-integrated, TDRKH excluded from feature selection)**

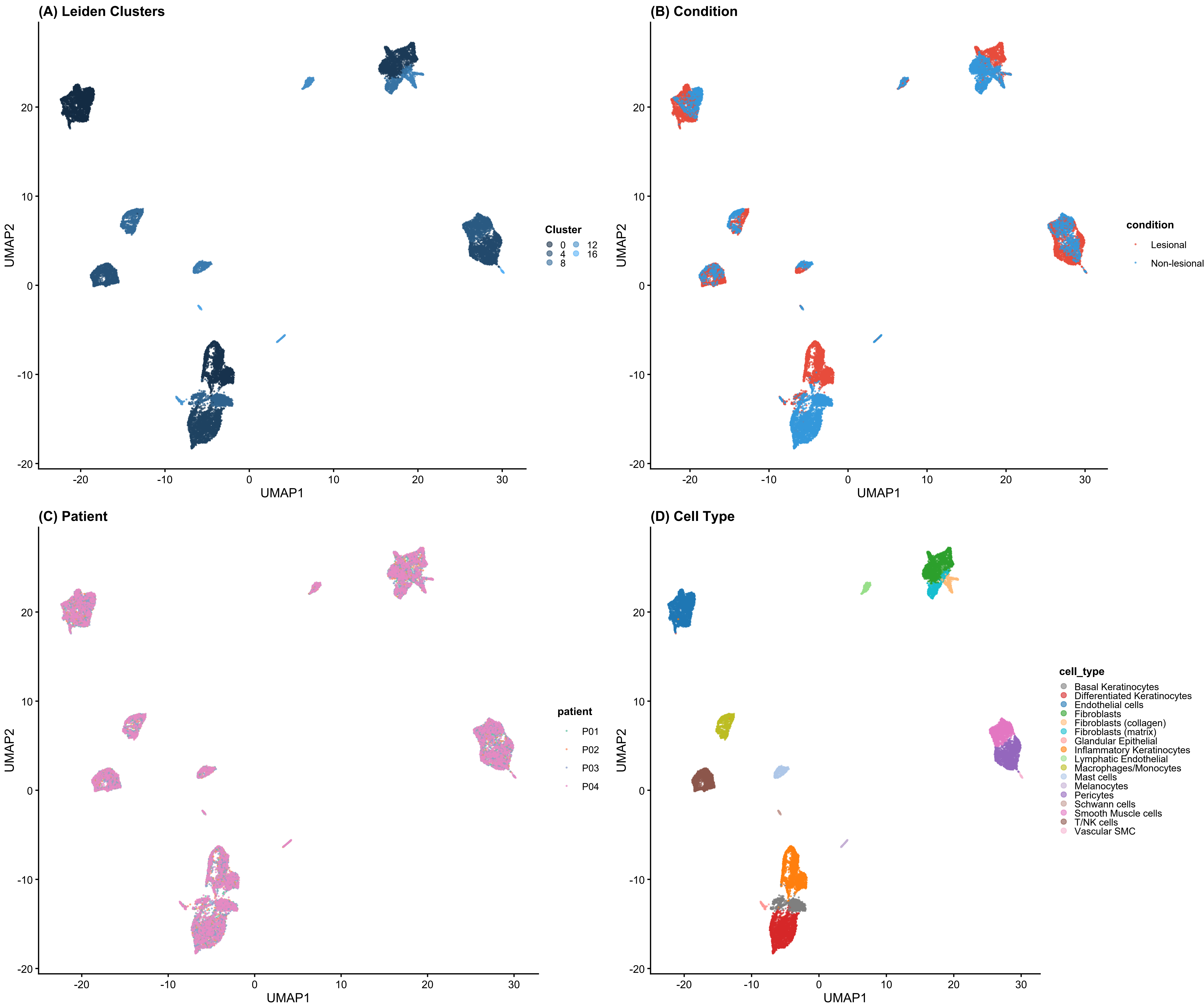

Supplement: Supplementary file 2 [file DataSheet2.pdf]
