# Supplementary material for: Post-transcriptional regulation via alternative polyadenylation and piRNA shapes macrophage responses in psoriasis
Source: Front Immunol. 2026 Jul 23;17:1760348. doi: 10.3389/fimmu.2026.1760348 (PMC13441757; doi:10.3389/fimmu.2026.1760348)
Supplement: Supplementary file 5 [file DataSheet5.pdf]

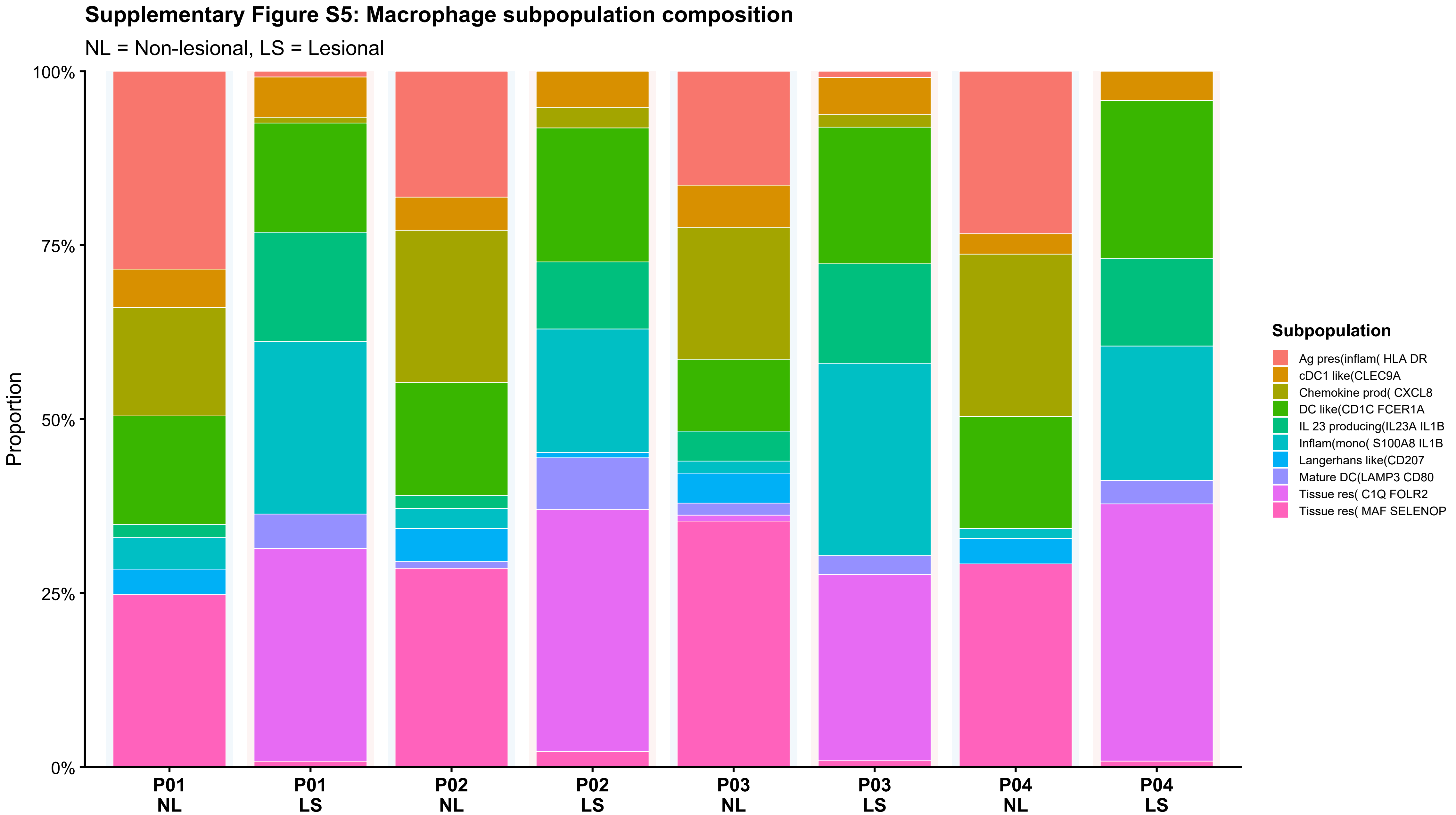

Supplementary Figure S5: Macrophage subpopulation composition
NL = Non-lesional, LS = Lesional
Proportion
100%
75%
50%
25%
0%
P01 NL
P01 LS
P02 NL
P02 LS
P03 NL
P03 LS
P04 NL
P04 LS
Subpopulation
Ag pres(inflam(HLA DR
cDC1 like(CLEC9A
Chemokine prod(CXCL8
DC like(CD1C FCER1A
IL 23 producing(IL23A IL1B
Inflam(mono(S100A8 IL1B
Langerhans like(CD207
Mature DC(LAMP3 CD80
Tissue res(C1Q FOLR2
Tissue res(MAF SELENOP
