# Supplementary material for: Post-transcriptional regulation via alternative polyadenylation and piRNA shapes macrophage responses in psoriasis
Source: Front Immunol. 2026 Jul 23;17:1760348. doi: 10.3389/fimmu.2026.1760348 (PMC13441757; doi:10.3389/fimmu.2026.1760348)

Supplementary Figure S6: Pathway activation scores

TDRKH-high vs TDRKH-low macrophages (Mann-Whitney U test, one-sided)

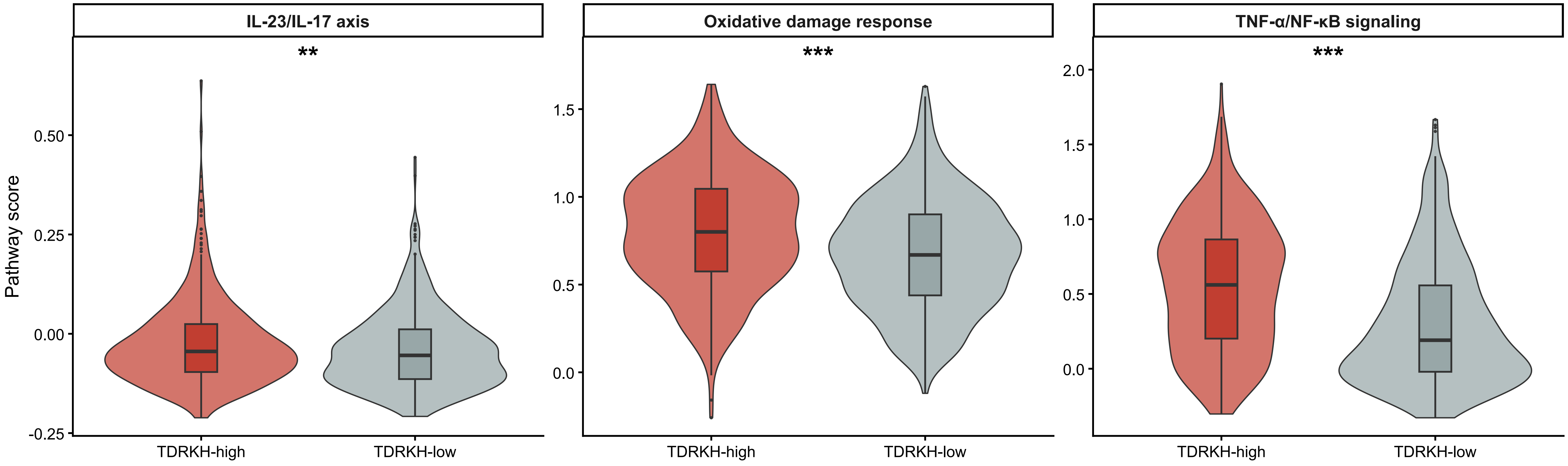

Supplement: Supplementary file 6 [file DataSheet6.pdf]
