# Supplementary material for: Post-transcriptional regulation via alternative polyadenylation and piRNA shapes macrophage responses in psoriasis
Source: Front Immunol. 2026 Jul 23;17:1760348. doi: 10.3389/fimmu.2026.1760348 (PMC13441757; doi:10.3389/fimmu.2026.1760348)

Supplementary Figure S7: Clustering robustness — TDRKH exclusion validation

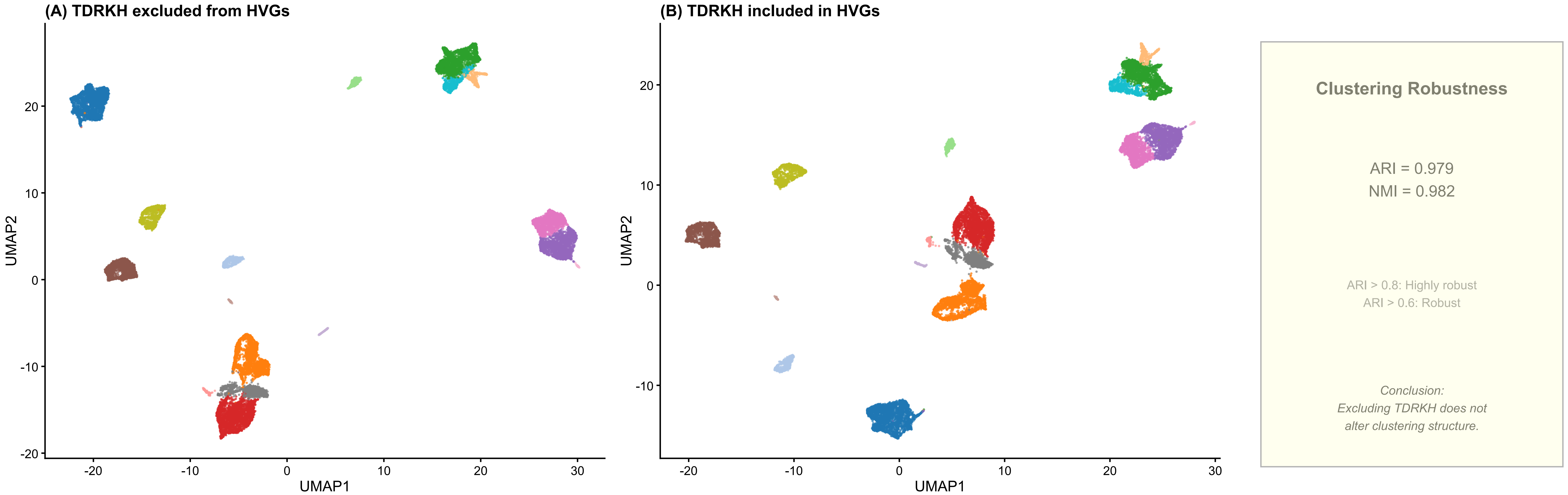

Supplement: Supplementary file 7 [file DataSheet7.pdf]
