# Supplementary material for: Post-transcriptional regulation via alternative polyadenylation and piRNA shapes macrophage responses in psoriasis
Source: Front Immunol. 2026 Jul 23;17:1760348. doi: 10.3389/fimmu.2026.1760348 (PMC13441757; doi:10.3389/fimmu.2026.1760348)
Supplement: Supplementary file 8 [file DataSheet8.pdf]

Supplementary Table S1: Macrophage subpopulation markers and literature references

Supplementary Table S1: Macrophage subpopulation markers and literature references

Subpopulation	Markers	Function	References
Tissue-res. (C1Q+FOLR2+)	C1QA, C1QB, FOLR2, LYVE1, MRC1, CD163	Complement-expressing, phagocytic, homeostatic	Mulder et al., Cell 2021<br>Kuznetsova et al., Nat Immunol 2020
Tissue-res. (MAF+SELENOP+)	MAF, SELENOP, SLC40A1, RNASE1	Anti-inflammatory, metabolic, protective	Dick et al., Nat Immunol 2022<br>Chakarov et al., Science 2019
DC-like (CD1C+FCER1A+)	CD1C, FCER1A, CLEC10A	cDC2, antigen presentation	Villani et al., Science 2017<br>Brown et al., Nat Immunol 2019
Inflam. mono. (S100A8+IL1B+)	S100A8, S100A9, IL1B, NAMPT, TYROBP	Alarmin-producing, pro-inflammatory	Zilionis et al., Immunity 2019<br>Molgora et al., Cell 2020
Ag-pres. inflam. (HLA-DR+)	HLA-DRA, HLA-DRB1, IL1R2, GPAT3	MHC-II antigen presentation	Lavin et al., Cell 2014<br>Park et al., Science 2020
Chemokine-prod. (CXCL8+)	CXCL8, CXCL2, NRP2, GPR183	Neutrophil/immune cell recruitment	Cheng et al., Cell 2021<br>de Oliveira et al., Nat Rev Immunol 2016
IL-23-producing (IL23A+IL1B+)	IL23A, IL1B, CXCL8, CD86, SERPINB1	IL-23/IL-17 axis driver; highest TDRKH	Gaffen et al., Nat Rev Immunol 2014<br>Lowes et al., Annu Rev Immunol 2014
cDC1-like (CLEC9A+)	CLEC9A, CADM1, DNASE1L3, WDFY4	cDC1, cross-presentation	Guilliams et al., Nat Rev Immunol 2014<br>Dutertre et al., Immunity 2019
Mature DC (LAMP3+CD80+)	LAMP3, CD80, MARCKSL1	Mature/activated DC, co-stimulatory	Maier et al., Sci Immunol 2020<br>Cheng et al., Cell 2021
Langerhans-like (CD207+)	CD207, HLA-DQB2, CLDN1, CD1A	Epidermal barrier immune surveillance	Clayton et al., Nat Rev Immunol 2017<br>Deckers et al., J Invest Dermatol 2018

Note: IL-23-producing subpopulation (highlighted) showed highest TDRKH expression and was enriched in lesional tissue.
